# Dentoskeletal effects of the maxillary splint headgear in the early correction of Class II malocclusion

**DOI:** 10.1186/s40510-020-00311-x

**Published:** 2020-05-04

**Authors:** José Augusto M. Miguel, Caterina Masucci, Luciana Quintanilha Pires Fernandes, Flavia Artese, Lorenzo Franchi, Veronica Giuntini

**Affiliations:** 1 Department of Orthodontics, School of Dentistry, State University of Rio de Janeiro, Boulevard 28 de Setembro, 157, Vila Isabel, Rio de Janeiro, RJ 20551-030 Brazil; 2 Sous-section Orthopédie Dento-Faciale, Faculté de Chirurgie dentaire, Université de Nice Sophia Antipolis, Nice, France; 3 Department of Experimental and Clinical Medicine, Section of Dentistry, Orthodontics, University of Florence, Florence, Italy

**Keywords:** Angle Class II, Extraoral traction appliance, Dental overjet, Incisor protrusion

## Abstract

**Background:**

To compare dentoskeletal changes produced by the maxillary splint headgear and cervical headgear appliance during the early phase of Class II treatment, specially the initial overjet and upper incisors position.

**Subjects and methods:**

In this retrospective study, 28 Class II patients treated with the maxillary splint headgear (MSG, mean age 10.1 ± 1.9 years) and 28 Class II patients treated with cervical headgear (CHG, mean age 9.5 ± 1.9 years) were evaluated before and after treatment. Statistical comparisons between the two groups for cephalometric measurements at T1 and for T2-T1 changes were performed by means of independent sample *t* tests.

**Results:**

The MSG showed a significantly greater reduction of the overjet in comparison to the CHG (− 2.4 mm and − 0.7 mm, respectively) and a significantly greater maxillary incisor uprighting (− 1.8 mm and 0.4 mm, respectively). In the MSG, overjet correction was due mainly to mandibular advancement (3.5 mm), while the correction of molar relationship (3.9 mm) was 64% skeletal and 36% dentoalveolar. In the CHG, the overjet correction was also more skeletal, due to mandibular growth (1.8 mm), while correction of molar relationship (3.5 mm) was 63% dentoalveolar and 37% skeletal.

**Conclusions:**

Both groups showed favorable skeletal mandibular changes, which was more significant in the MSG. Regarding tooth movement, the maxillary splint headgear was more effective in uprighting upper incisors and reducing the overjet than cervical headgear appliance.

## Introduction

Early treatment of Class II consists of two phases of treatment, with the objective of improving patient’s growth pattern and preventing risks associated with this malocclusion [1]. However, studies evaluating long-term dentoskeletal changes show that there is no difference in the effectiveness (the ability to be successful and produce the intended results) of Class II correction when compared to one- or two-phase treatments [1–3]. Therefore, early treatment is considered less efficient (the ability to produce the intended results without any waste) than late treatment, since it increases total orthodontic treatment time and has additional cost and burden to the patient, parent, and clinician [1, 2, 4].

A Cochrane systematic review concluded that early treatment of Class II has the advantage of reducing the incidence of trauma of proclined upper incisors when compared to late treatment, independent of the type of orthodontic appliance used [5]. Another systematic review, published by Nguyen et al. [6] found that patients with an overjet greater than 3 mm are approximately twice as much at risk of trauma to anterior teeth than patients with an overjet smaller than 3 mm. Petti [7] performed a meta-analysis and observed that the proportion of dental trauma on permanent teeth in people with an overjet of 3 to 4 mm and 5 to 7 mm that are attributable to these overjets is 21.8% and 10.2%, respectively.

Another reason for early treatment of Class II is the possibility of bullying among children, since teeth are the characteristic most frequently targeted for bullying, and proclined upper incisors are one of the three most commonly reported dentofacial features targeted by bullies [8]. Besides that, Al-Omari et al. [9] reported that 12-year-old bullied children have more negative impact on their oral health-related quality of life than children who did not report being bullied. So, for children who are bullied because of dentofacial characteristics, orthodontic treatment can have an important impact on their oral health-related quality of life. Therefore, to justify the early treatment of Class II, the first phase of treatment should aim at reducing the overjet and uprighting the proclined upper incisors, in order to reduce the incidence of dental trauma and protect patients from possible bullying.

The cervical headgear commonly used for Class II correction in the first phase of treatment is aimed at restricting the anterior maxillary growth [10] by applying an antero-posterior force on the upper first molars [11]. When evaluating the dental changes produced by this kind of headgear, a predominance of distalization and inclination of the supporting teeth is observed [12, 13]. Mantysaari et al. [14] reported that the headgear was able to restrict maxillary growth (SNA = − 1.7°) and improve the Class II skeletal relationship (ANB = − 2.6°); however, the overjet was not altered, and the upper incisors remained proclined (IU/SN = 4.7°).

Raymond Thurow, in 1975, described another extraoral appliance for the correction of Class II malocclusion, which consists of an acrylic maxillary splint covering all upper teeth and the palate associated with a high-pull traction known as the Thurow appliance or maxillary splint headgear. Thus, the first molars are no longer the only anchorage unit of the appliance. Therefore, larger effects are obtained also on upper incisors, since an “en masse” movement of all the teeth included in the splint occurs [12]. It is also a versatile appliance allowing for modifications of its original design such as the inclusion of an expansion screw [15, 16] or a palatal crib [17, 18], if the patient presents with a constricted upper arch or an open bite, respectively. Teuscher [19] proposed an appliance similar to the maxillary splint headgear associated to a functional appliance with the purpose of including a mandibular advancement effect. Fernandes et al. [20] reported an uprighting of upper (1-NA = − 3.4 mm) and lower (1-NB = − 1.4 mm) incisors and an improvement of Class II skeletal relationship (ANB = − 0.8°) after treatment with the maxillary splint headgear. Caldwell et al. [21] found similar results and they also observed a 4.2 mm reduction of the overjet.

Few studies in the literature have evaluated the effects of the maxillary splint headgear [20, 21] and, to the best of our knowledge, no study compared them to the effects produced by the cervical headgear, the standard of care for this treatment. Therefore, the aim of this study was to compare the dentoskeletal effects of both appliances, specially the position of upper incisors and initial overjet, during the early phase of Class II treatment.

## Subjects and methods

This study was approved by the Ethical Committee of the Rio de Janeiro State University (number 2.281.471). It is a retrospective cephalometric study designed to compare the dentoskeletal effects produced by two treatment modalities for the correction of Class II malocclusion: the maxillary splint headgear and the cervical headgear appliances. Sample size calculation determined that for the independent *t* test, with a minimal detectable difference of 1.5 mm for the overjet, a standard deviation of 1.4 mm [10], an alpha level of 0.05, and a power of 0.80, 15 subjects were required for each group (BioEstat 5.0).

The inclusion criteria were as follows:

- Class II dentoskeletal relationships (ANB angle greater than 4 degrees);
- Overjet larger than 4 mm;
- Class II molar relationship;
- Absence of craniofacial anomalies (e.g., cleft lip and/or palate).

Lateral cephalograms for all subjects had to be available at the beginning of treatment (T1) and at the end of early treatment with an extraoral appliance (T2). Dental casts for the evaluation of the phase of the dentition had to be available at T1. Phase of the dentition was defined as follows [22]:

1. Early mixed dentition: Shedding of the deciduous incisors, eruption of the first permanent molars and permanent incisors;
2. Intermediate mixed dentition: Permanent incisors and first molars fully erupted, presence of all deciduous teeth in the buccal region (deciduous canine, first molar, and second molar);
3. Late mixed dentition: Shedding of one or more deciduous canines and molars, eruption of the permanent canines and premolars;
4. Early permanent dentition: Presence of all permanent teeth (possible presence of second molars; absence of third molars).

If the patient had some early loss of deciduous teeth, the stages of permanent teeth eruption were considered for classification.

All patients treated consecutively with maxillary splint headgear were derived from one private practice in Rio de Janeiro, while the cervical headgear group was from the records of the patients treated consecutively at the University of Florence. The maxillary splint headgear group (MSG) consisted of 28 patients (16 females and 12 males), and the cervical headgear group (CHG) included 28 patients (15 females and 13 males). During the observational period, no other appliances were used in both groups.

**Fig. 1:**
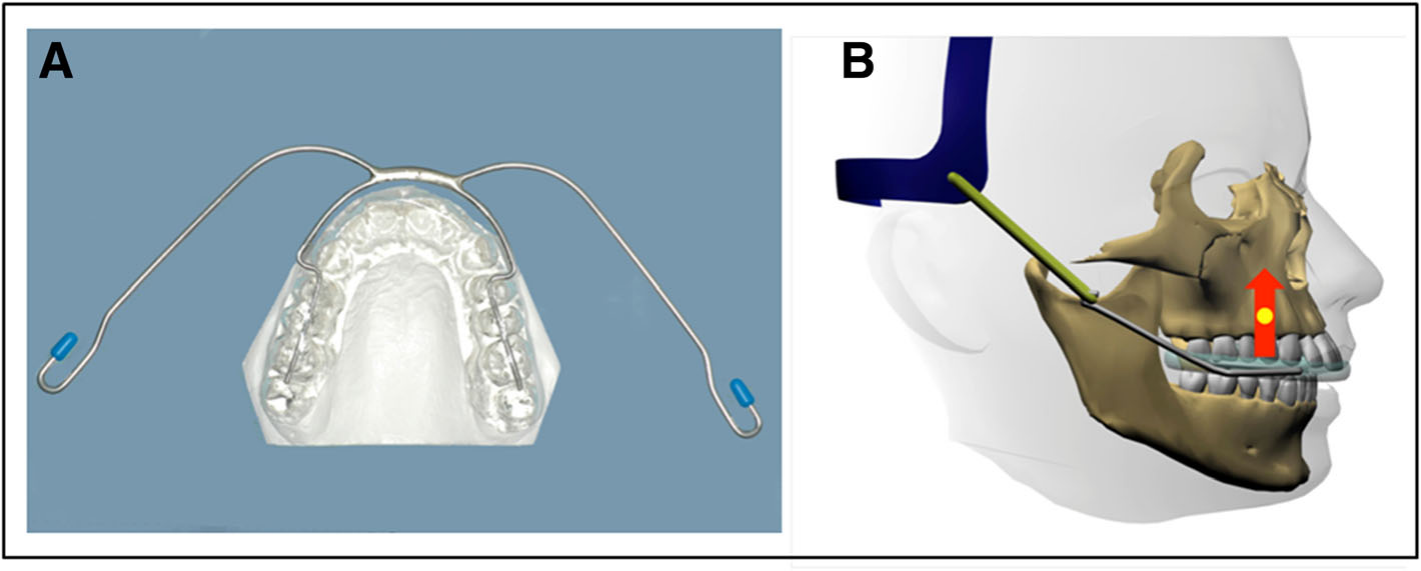
Maxillary splint headgear: A, occlusal aspect; B, arrow indicates the direction of high-pull extraoral traction

The maxillary splint headgear comprised a removable maxillary self-polymerizing acrylic resin splint extended laterally and occlusally, covering the cusps and approximately one third of the buccal surfaces of all teeth, and a high-pull orthopedic traction. The face bow was bent upwards at about 45° from the horizontal plane in relation to the intraoral arch and connected to an elastic (0.5 in. × 1.5 mm), which was attached to a head strap (Morelli Ortodontia Sorocaba, Brazil) (Fig. 1). The extraoral force was 400 g per side and it was calibrated with a dynamometer (Ohaus Corp., Florham Park, New Jersey, USA). Treatment with the maxillary splint headgear lasted 1.5 years on average. In the cervical headgear group, the upper first molars were banded, and a cervical orthopedic traction was applied. The face bow was bent upwards about 10° from the horizontal plane in relation to the intraoral arch. The cervical headgear delivered forces of 250 g per side that were calibrated with a dynamometer inserted in the spring modules (Leone Orthodontics Products, Sesto Fiorentino, Firenze). Treatment with cervical headgear lasted 1.7 years on average. Patients were instructed to wear both appliances for 14 h/day. During treatment, all patients were scheduled monthly so that the appliances could be adjusted, if necessary, and the forces could be verified. According to the patient’s records, if the patient had failed any appointment, this was re-scheduled as soon as possible and attendance was considered adequate for both groups. In the maxillary splint headgear, in cases of eruption of permanent canines and premolars, the acrylic resin was ground in order to obtain space for eruption.

### Cephalometric analysis

**Fig. 2:**
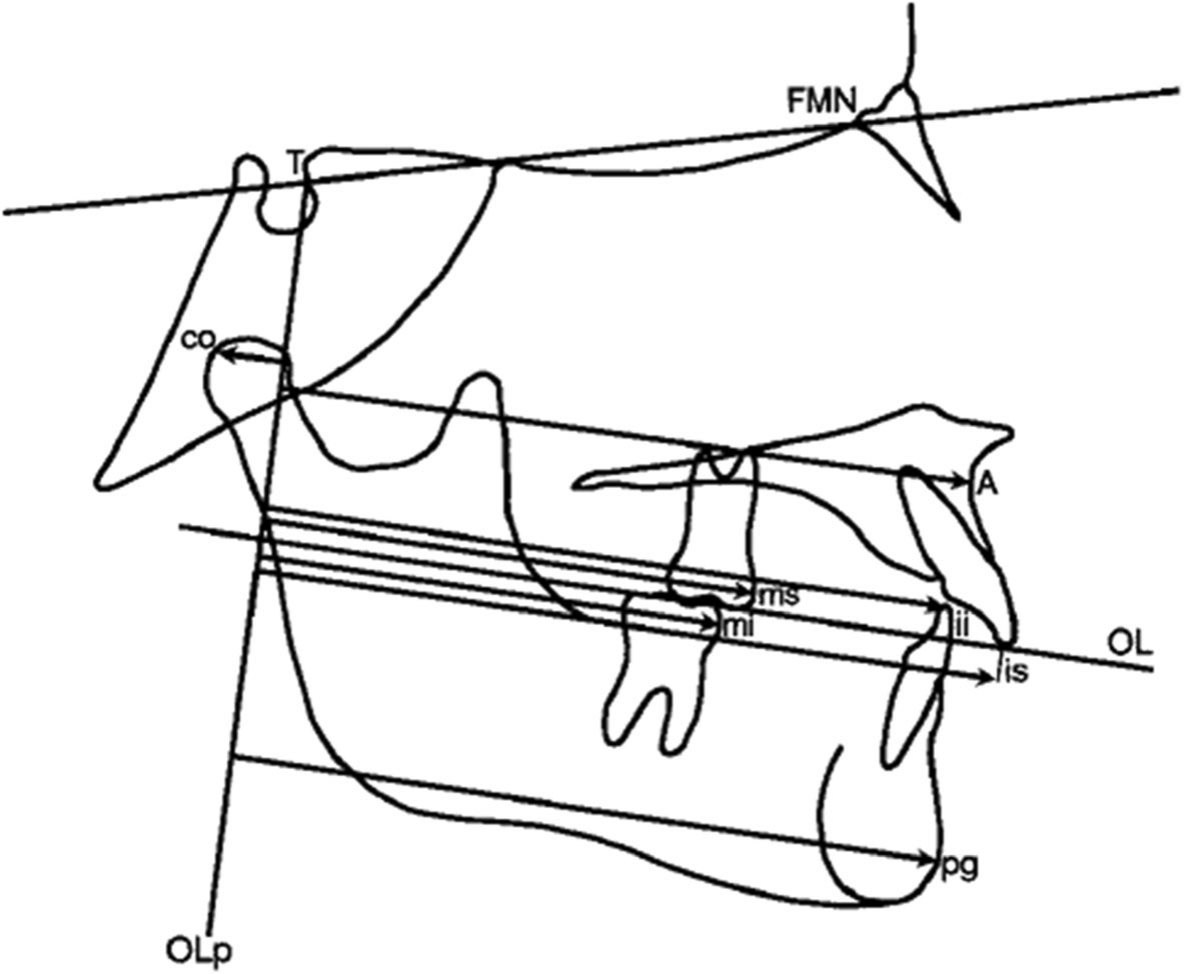
Modified Pancherz’s analysis

All lateral cephalograms were digitized and a customized cephalometric analysis was provided by a specific cephalometric software (Viewbox, version 3.0, dHAL Software, Kifissia, Greece). Fourteen variables (3 angular and 11 linear) were assessed for each tracing, according to a modified Pancherz`s cephalometric analysis (Fig. 2) [23]. The established enlargement factor for all cephalograms was standardized to a magnification factor of 0%.

All lateral cephalograms were traced initially by the same operator (J.M.) and were checked by a second operator (L.F.) to verify anatomical outlines, landmark placement, and superimposition. Both operators were blinded with regard to the origin of the headfilms and the group to which the patient belonged. Any disagreements were resolved to the satisfaction of both operators.

### Method error and statistical analysis

In order to determine the method error, the same operator redigitized twenty randomly selected cephalograms and recalculated the variables after 15 days. The systematic error was evaluated with the Wilcoxon signed-rank test. The random error was calculated with Springate’s method of moments’ estimator (MME) [24].

The Shapiro Wilk’s test showed normal distribution of cephalometric data at T1 and of T2-T1 changes. Therefore, statistical comparisons between the two groups for the cephalometric measurements at T1 (starting forms) and for the T2-T1 changes were performed by means of independent sample *t* tests. The difference in the distribution of the phases of the dentition in the 2 groups at T1 was assessed with Fisher Exact Probability Test (SPSS version 12.0, SPSS Inc., Chicago, IL).

## Results

Descriptive statistics and statistical comparisons for age and dentoskeletal features at T1 of the MSG and CHG were reported in Table 1. No statistically significant differences were found between the two groups as to age distribution (age of MSG 10.1 ± 1.9 years and age of CHG 9.5 ± 1.9 years) and cephalometric variables at T1 (starting forms). No significant difference in the distribution of the phases of the dentition at T1 was assessed (early mixed dentition 7.1% in MSG and 14.3% in CHG; intermediate mixed dentition 39.3% in MSG and 42.8% in CHG; late mixed dentition 39.3% in MSG and 42.8% in CHG; permanent dentition 14.3% in MSG and 0.0% in CHG; *p* = 0.206). The MME random error measurements ranged from 0.14 to 0.48. For the systematic error, the *p* values varied from 0.156 to 0.955 (Table 2).

**Table 1:** Descriptive statistics and statistical comparisons (independent sample *t* tests) for the cephalometric variables and age at T1 (starting forms)

	Maxillary splint headgear group (*N* = 28)		Cervical headgear group (*N* = 28)		Diff.	*P*	95% confidence interval	
	Mean	SD	Mean	SD			Lower	Upper
**Age (years)**	10.1	1.9	9.5	1.9	0.6	0.306	− 0.5	1.6
**Wits (mm)**	2.5	2.5	1.7	2.3	0.8	0.176	− 0.4	2.2
**Overjet (mm)**	7.0	2.0	6.2	1.8	0.8	0.101	− 0.2	1.8
**Molar relation (mm)**	1.0	1.6	1.4	1.3	− 0.4	0.356	− 1.2	0.4
**Maxillary base (A point-OLp) (mm)**	72.0	5.0	71.2	3.0	0.8	0.514	− 1.5	2.9
**Mandibular base (pg-OLp) (mm)**	71.0	5.4	72.2	4.5	− 1.2	0.358	− 3.9	1.4
**Condylar head (co-OLp) (mm)**	7.9	2.2	7.6	2.7	0.3	0.665	− 1.1	1.6
**Mandibular length (pg-OLp+co-OLp) (mm)**	78.9	5.7	79.8	4.6	− 0.9	0.493	− 3.7	1.8
**Maxillary incisor (is-OLp) (mm)**	80.9	6.2	78.3	3.8	2.6	0.067	− 0.2	5.3
**Mandibular incisor (ii-OLp) (mm)**	73.9	5.8	72.1	3.6	1.8	0.184	− 0.8	4.3
**Maxillary molar (ms-OLp) (mm)**	49.8	4.8	48.3	3.1	1.5	0.169	− 0.7	3.7
**Mandibular molar (mi-OLp) (mm)**	48.8	5.5	46.9	3.9	1.9	0.144	− 0.7	4.4
**Overbite (mm)**	1.9	2.1	2.9	2.2	− 1.0	0.074	− 2.2	0.1
**SN to Pal.Pl. (deg.)**	7.0	3.2	7.9	3.0	− 0.9	0.304	− 2.5	0.8
**SN to Mand. Pl. (deg.)**	35.3	5.4	34.4	5.6	0.9	0.542	− 2.0	3.8
**Pal. Pl. to Mand. Pl. (deg.)**	28.3	4.4	26.5	5.7	1.8	0.198	− 1.0	4.5

*Diff*. difference, *deg*. degrees, *Pal*. palatal, *Pl*. Plane, *Mand*. mandibular

**Table 2:** Random and systematic errors for the cephalometric variables

Variable	Random error (MME)	Systematic error (*P* values)
**Wits (mm)**	1.09	0.558
**Overjet (mm)**	0.22	0.363
**Molar relation (mm)**	0.14	0.156
**Maxillary base (A point-OLp) (mm)**	0.22	0.307
**Mandibular base (pg-OLp) (mm)**	0.34	0.955
**Condylar head (co-OLp) (mm)**	0.37	0.460
**Mandibular length (pg-OLp+co-OLp) (mm)**	0.48	0.820
**Maxillary incisor (is-OLp) (mm)**	0.32	0.733
**Mandibular incisor (ii-OLp) (mm)**	0.26	0.865
**Maxillary molar (ms-OLp) (mm)**	0.37	0.820
**Mandibular molar (mi-OLp) (mm)**	0.34	0.733
**Overbite (mm)**	0.18	0.532
**SN to Pal.Pl. (deg.)**	0.25	0.865
**SN to Mand. Pl. (deg.)**	0.38	0.820
**Pal. Pl. to Mand. Pl. (deg.)**	0.38	0.820

**Fig. 3:**
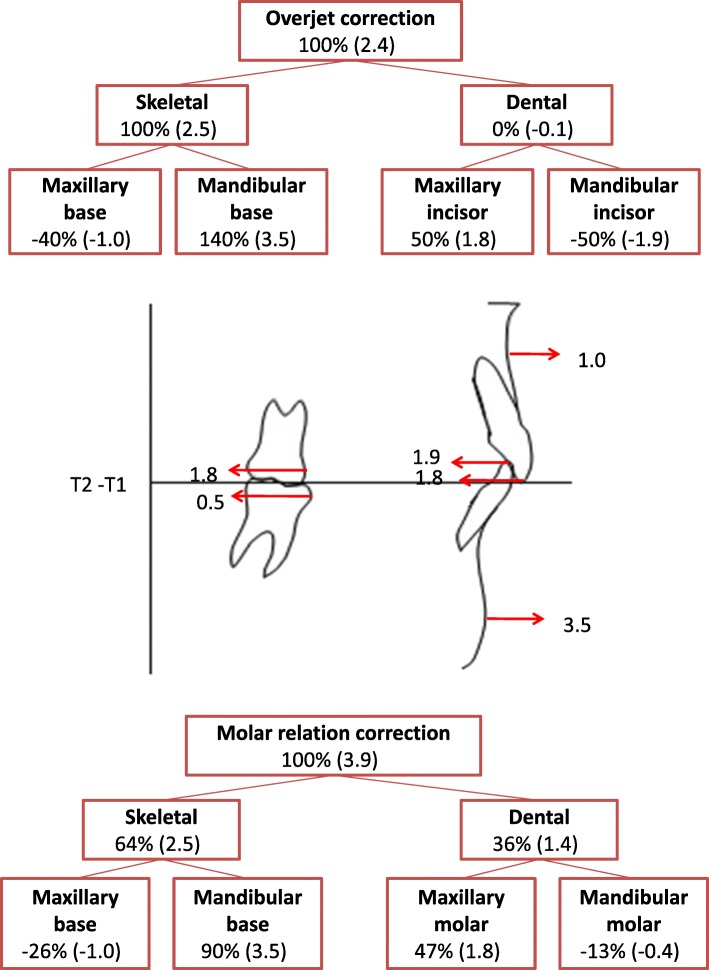
Maxillary and mandibular skeletal and dentoalveolar changes contributing to sagittal overjet correction and molar correction during the treatment period with maxillary splint headgear

**Fig. 4:**
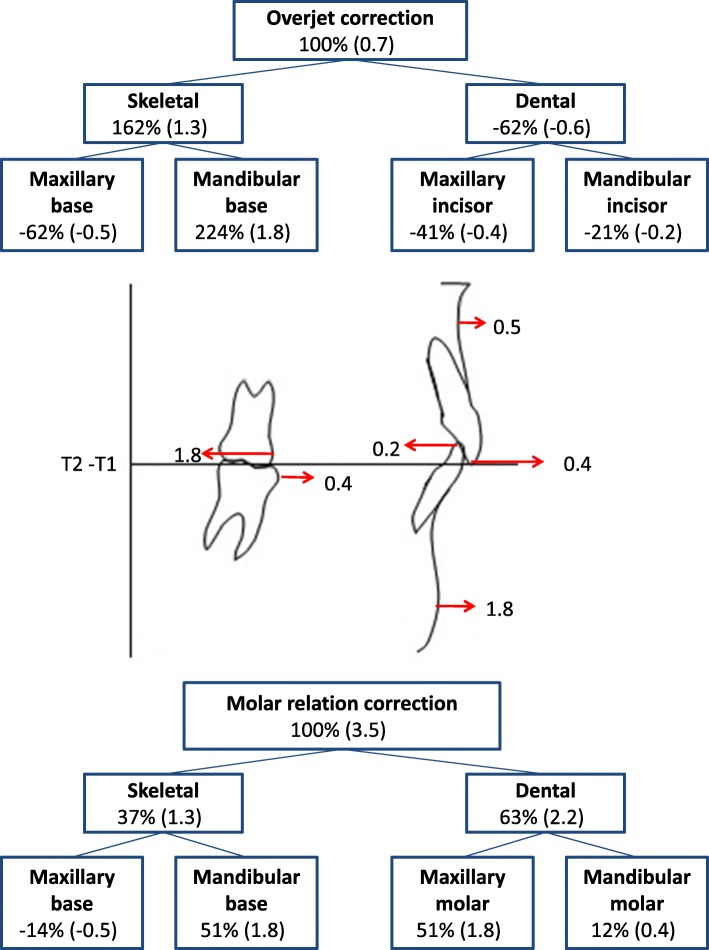
Maxillary and mandibular skeletal and dentoalveolar changes contributing to sagittal overjet correction and molar correction during the treatment period with cervical headgear appliance

Table 3 reported the statistical comparison between the T1-T2 changes for the two treatment groups. The overjet showed a significantly greater decrease in the MSG (− 2.4 mm) with respect to the CHG (− 0.7 mm). In both groups, the amount of overjet correction was associated with favorable skeletal changes in the mandibular base (Figs. 3 and 4). In particular, in the MSG, the 2.4 mm of overjet correction was due mainly to 3.5 mm of mandibular advancement and 1.0 mm of maxillary advancement (Fig. 3). In the CHG (Fig. 4), the 0.7 mm of overjet correction was due to 1.3 mm of favorable skeletal changes (derived from the combination of 1.8 mm of mandibular advancement and 0.5 of maxillary advancement) and 0.6 mm of unfavorable dental changes (consisting of 0.4 mm of proclination of the upper incisors and 0.2 mm of retroclination of the lower incisors).

**Table 3:** Descriptive statistics and statistical comparisons (independent sample *t* tests) for the T2-T1 changes

	Maxillary splint headgear group (*N* = 28)		Cervical headgear group (*N* = 28)		Diff.	*P*	95% confidence interval	
	Mean	SD	Mean	SD			Lower	Upper
**Age (years)**	1.6	0.6	1.7	0.6	− 0.1	0.339	− 0.5	0.2
**Wits (mm)**	− 0.6	2.2	− 0.2	3.2	− 0.4	0.604	− 1.9	1.1
**Overjet (mm)**	− 2.5	1.9	− 0.8	0.9	− 1.7	**0.000**	− 2.5	− 0.9
**Molar relation (mm)**	− 3.9	2.0	− 3.5	1.5	− 0.4	0.428	− 1.3	0.6
**Maxillary base (A point-OLp) (mm)**	1.0	2.0	0.5	2.0	0.5	0.326	− 0.5	1.6
**Mandibular base (pg-OLp) (mm)**	3.5	4.0	1.8	2.6	1.7	0.057	− 0.1	3.6
**Condylar head (co-OLp) (mm)**	− 0.1	1.9	0.7	1.3	− 0.8	0.051	− 1.7	0.0
**Mandibular length (pg-OLp+co-OLp) (mm)**	3.4	3.3	2.5	2.3	0.9	0.260	− 0.7	2.4
**Maxillary incisor (is-OLp minus A point-OLp) (mm)**	− 1.8	1.4	0.4	1.4	− 2.2	**0.000**	− 3.0	− 1.5
**Mandibular incisor (ii-OLp minus pg-OLp) (mm)**	− 1.9	1.5	− 0.2	1.2	− 1.7	**0.000**	− 2.5	− 1.0
**Maxillary molar (ms-OLp minus A point-OLp) (mm)**	− 1.8	1.9	− 1.8	1.8	0.0	0.997	− 1.0	1.0
**Mandibular molar (mi-OLp minus pg-OLp) (mm)**	− 0.5	1.4	0.4	1.5	− 0.9	0.034	− 1.6	− 0.1
**Overbite (mm)**	0.2	1.9	0.0	1.7	0.2	0.642	− 0.7	1.2
**SN to Pal.Pl. (deg.)**	1.2	1.3	1.0	1.2	0.2	0.622	− 0.5	0.9
**SN to Mand. Pl. (deg.)**	0.3	1.4	− 0.6	1.8	0.9	0.051	0.0	1.7
**Pal. Pl. to Mand. Pl. (deg.)**	− 0.9	1.5	− 1.6	2.1	0.7	0.180	− 0.3	1.7

*Diff*. difference, *deg*. degrees, *Pal*. palatal, *Pl*. plane, *Mand*. mandibular

The amount of molar correction (3.9 mm and 3.5 mm, respectively) was not significantly different between the MSG and CHG. In the MSG, about two thirds of molar correction (2.5 mm) were due to skeletal changes (obtained by the combination of 3.5 mm of mandibular advancement and to 1.0 mm of maxillary advancement) and about one third of the correction (1.4 mm) was due to dental changes (obtained by the combination of 1.8 mm of maxillary molar distalization and 0.4 mm of mandibular molar distalization) (Fig. 3). On the other hand, in the CHG (Fig. 4) about two thirds of molar correction (2.2 mm) were due to dental changes (achieved by means of 1.8 mm of maxillary molar distalization and 0.4 mm of mandibular molar mesialization) and about one third of the correction (1.3 mm) was due to skeletal changes (derived from the combination of 1.8 mm of mandibular advancement and 0.5 mm of maxillary advancement).

As for the skeletal changes, the maxillary advancement was not significantly different between MSG and CHG (maxillary base 1.0 mm and 0.5 mm, respectively). In regard the mandible, both groups showed a favorable mandibular advancement (mandibular base 3.5 mm and 1.8 mm in the MSG and CHG, respectively) and an increase in mandibular length (3.4 mm and 2.5 mm in the MSG and CHG, respectively), although no statistically significant between-group differences were found. The sagittal position of the condyle (condylar head) remained almost unchanged in both groups.

As for the dentoalveolar changes, the MSG showed a significantly greater retroclination of the maxillary incisors when compared to the CHG (is-OLp minus A point-OLp − 1.8 mm and 0.4 mm, respectively). The mandibular incisor exhibited a significantly greater retroclination in the MSG with respect to CHG (ii-OLp minus pg-OLp − 1.9 mm and − 0.2 mm, respectively). No statistically significant differences between the 2 groups were found for maxillary molar position (ms-OLp minus A point-OLp − 1.8 mm in both groups) and mandibular molar position (mi-OLp minus pg-OLp − 0.5 mm and 0.4 mm in MSG and CHG, respectively) and for overbite (0.2 mm and 0 mm in MSG and CHG, respectively). Also, no statistically between-group differences were found for any of the variables describing the skeletal vertical relationships. Both groups showed a slight decrease in the intermaxillary divergence (Palatal Plane to Mandibular Plane angle).

## Discussion

This study compared the dentoskeletal effects produced by the cervical headgear and the maxillary splint headgear. Considering that the present sample was in an active growth period, the results found should be attributed to a combined effect of growth and appliance effect. Although patients were instructed to wear the appliance for the same amount of time in both groups (14 h/day), the force applied was different between them, since in the MSG the force was distributed along more teeth, allowing the use of heavier forces (400 g per side) when comparing to the CHG force (250 g per side), which was anchored only on the first permanent molars. The modified Pancherz’s cephalometric analysis used in the present study was chosen mainly for two reasons: (1) It was close to the investigated area; (2) the points and lines used as a reference do not undergo any significant remodeling during growth, making it possible to evaluate the interrelationship between skeletal and dental changes in and between the two jaws [25].

The MSG showed a greater overjet reduction (− 2.4 mm) than the CHG (− 0.7 mm). Caldwell et al. [21], comparing patients treated with the maxillary splint headgear with an untreated control group, observed an overjet reduction of 4.2 mm and 0.5 mm, respectively, demonstrating that the maxillary splint headgear is able to reduce significantly the overjet. Tulloch et al. [10] observed a 1.5-mm overjet reduction in patients treated with cervical headgear, while there were no changes in the control group. In this study, the maxillary splint headgear allowed for greater overjet reduction than the cervical headgear. Interestingly, in both groups, the overjet improvement was due primarily to the mandibular growth (3.5 mm and 1.8 mm, in the MSG and CHG, respectively). It may be implied that in the MSG, the disocclusion caused by the maxillary splint could have allowed a greater mandibular growth in these patients. The MSG also showed a significantly greater retraction of upper incisors (− 1.8 mm) than the CHG that actually exhibited a slight projection of these teeth (0.4 mm). Similar results demonstrating upper incisor retroclination with the use of the maxillary splint headgear have been described in the literature [15, 20], as well as the slight upper incisor proclination in patients treated with cervical headgear [14, 26]. These outcomes obtained in MSG can be explained by use of the maxillary splint covering the upper incisors and a high-pull traction, which allow the transmission of retraction and intrusion forces to these teeth. On the other hand, in the CHG, the cervical headgear arch was positioned at a 2-mm distance from the upper incisors, thus preventing the upper lip to contact these teeth [14]. A greater amount of lower incisors retraction was observed in the MSG (− 1.9 mm) when compared to the CHG (− 0.2 mm). Fernandes et al. [20] also found a retroclination of lower incisors after treatment with the maxillary splint headgear (− 1.4 mm).

Molar correction was similar in both groups (3.9 mm in the MSG and 3.5 mm in the CHG), as well as upper molar distalization (1.8 mm in the MSG and CHG). Fernandes et al. [20] found a smaller amount of distal movement of upper molars after maxillary splint headgear use (0.7 mm).

In the present study, none of the tested appliances produced significant changes in overbite. On the contrary, Caldwell et al. [21] observed 2 mm of overbite reduction, which was due especially to the lower arch leveling in patients treated with the maxillary splint headgear.

In regard the skeletal changes, a small maxillary advancement was seen in both groups, even though clinically irrelevant (1.0 mm in the MSG and 0.5 mm in the CHG). Since patients were growing, a restriction of maxillary advancement could be expected instead of a retraction of maxilla. Fernandes et al. [20] observed that after treatment with the maxillary splint headgear maxillary growth restriction occurred (SNA = − 0.7°) and Tulloch et al. [10] found similar results with the use of the cervical headgear (SNA = − 0.9°). The increase in mandibular length found in this study in the MSG (3.4 mm) was greater than that described by Fernandes et al. [20] (Co-Gn = 1.8 mm). Although there was no statistically significant difference in the mandibular forward movement between groups (3.5 mm in the MSG and 1.8 mm in the CHG), greater displacement was observed in the MSG. This could be justified since the mandible in these patients was more free to move without occlusal interference, and this could be considered an advantage of the maxillary splint headgear. In relation to the vertical changes, none of the groups presented significant modifications.

This study has some limitations, such as the retrospective study design and the absence of a control group due to ethical reasons. In addition, the incidence of incisor trauma or injuries, before and after orthodontic treatment, were not evaluated, as well as the psychosocial impact of early treatment, due to the retrospective nature of the study. So, other prospective studies are suggested evaluating, apart from the dentoalveolar and skeletal effects of headgears, the incidence of dental trauma and the psychosocial impact that treatment in the mixed dentition may cause in Class II patients. Since previous studies showed that overjet reduction in children may reduce the risk of trauma in anterior teeth [5, 6] and proclined upper incisors are an important reported dentofacial feature targeted by bullies [9], the maxillary splint headgear should be considered as an effective option for Class II early treatment, in cases that overjet reduction and upper incisors uprighting are desired.

## Conclusions

Patients in MSG and CHG showed favorable skeletal mandibular changes and a slight maxillary advancement. Since the maxillary splint headgear was able to upright the upper incisors and produce a greater reduction of the overjet, it can be considered an effective alternative to early treatment of Class II patients with proclined upper incisor and increased overjet, rather than the cervical headgear.

## Data Availability

The datasets used and/or analyzed during the current study are available from the corresponding author on reasonable request.

## Abbreviations

CHG: Cervical headgear group
MSG: Maxillary splint headgear
MME: Method of moments’ estimator
